# The Temple University Hospital Seizure Detection Corpus

**DOI:** 10.3389/fninf.2018.00083

**Published:** 2018-11-14

**Authors:** Vinit Shah, Eva von Weltin, Silvia Lopez, James Riley McHugh, Lillian Veloso, Meysam Golmohammadi, Iyad Obeid, Joseph Picone

**Affiliations:** Department of Electrical and Computer Engineering, Temple University, Philadelphia, PA, United States

**Keywords:** EEG, electroencephalogram, seizure detection, machine learning, annotated data, Temporal-Spatial sequence data, automatic seizure detection

## Introduction

The electroencephalogram (EEG), which has been in clinical use for over 70 years, is still an essential tool for diagnosis of neural functioning (Kennett, 2012). Well-known applications of EEGs include identification of epilepsy and epileptic seizures, anoxic and hypoxic damage to the brain, and identification of neural disorders such as hemorrhagic stroke, ischemia and toxic metabolic encephalopathy (Drury, 1988). More recently there has been interest in diagnosing Alzheimer's (Tsolaki et al., 2014), head trauma (Rapp et al., 2015), and sleep disorders (Younes, 2017). Many of these clinical applications now involve the collection of large amounts of data (e.g., 72-h continuous EEG recordings), which makes manual interpretation challenging. Similarly, the increased use of EEGs in critical care has created a significant demand for high-performance automatic interpretation software (e.g., real-time seizure detection).

A critical obstacle in the development of machine learning (ML) technology for these applications is the lack of big data resources to support training of complex deep learning systems. One of the most popular transcribed seizure databases available to the research community, the CHB-MIT Corpus (Goldberger et al., 2000), only consists of 23 subjects. Though high performance has been achieved on this corpus (Shoeb and Guttag, 2010), these results have not been representative of clinical performance (Golmohammadi et al., 2018). Therefore, we introduce the TUH EEG Seizure Corpus (TUSZ), which is the largest open source corpus of its type and represents an accurate characterization of clinical conditions.

Since seizures occur only a small fraction of the time in this type of data, and manual annotation of such low-yield data would be prohibitively expensive and unproductive, we developed a triage process for locating seizure recordings. We automatically selected data from the much larger TUH EEG Corpus (Obeid and Picone, 2016) that met certain selection criteria. Three approaches were used to identify files with a high probability that a seizure event occurred: (1) keyword search of EEG reports for sessions that were likely to contain seizures (e.g., reports containing phrases such as “seizure begins with” and “evolution”), (2) automatic detection of seizure events using commercially available software (Persyst Development Corporation., 2017), and (3) automatic detection using an experimental deep learning system (Golmohammadi et al., 2018). Data for which approaches (2) and (3) were in agreement were given highest priority.

Accurate annotation of an EEG requires extensive training. For this reason, manual annotation of EEGs is usually done by board-certified neurologists with many years of post-medical school training. Consequently, it is difficult to transcribe large amounts of data because such expertise is in short supply and is most often focused on clinical practice. Previous attempts to employ panels of experts or use crowdsourcing strategies were not productive (Obeid et al., 2017). However, we have demonstrated that a viable alternative is to use a team of highly trained undergraduates at the Neural Engineering Data Consortium (NEDC) at Temple University. These students have been trained to transcribe data for seizure events (e.g., start/stop times; seizure type) at accuracy levels that rival expert neurologists at a fraction of the cost (Obeid et al., 2017; Shah et al. in review). In order to validate the team's work, a portion of their annotations were compared to those of expert neurologists and shown to have a high inter-rater agreement.

In this paper, we describe the techniques used to develop TUSZ, evaluate their effectiveness, and present some descriptive statistics on the resulting corpus.

## Methods

To build an annotated seizure dataset, we first needed an abundant source of EEG data. Our work here utilized a subset which includes approximately 90% of v0.6.0 of TUH EEG. The data is organized by patient and by session. Each session contains EEG signal data stored in a standard European Data Format (EDF; Kemp, 2013) and a de-identified report written by a board-certified neurologist. The EDF files contain a variable number of channels (Obeid and Picone, 2016) but during the annotation process only 19 EEG channels plus two supplementary channels (heart rate and photic stimulation) were used. The data were annotated using our open source annotation tool (Capp et al., 2017).

Since <0.1% of the original data contains actual seizure events, annotating the entire database would be costly and inefficient. Therefore, we used three independent approaches to identify sessions that were likely to contain actual seizure events. First, we applied off-the-shelf natural language processing (NLP) techniques to identify reports that had keywords related to ictal patterns. The reports were preprocessed using filters that normalized (e.g., removed punctuation and misspellings) and stemmed the text (Sirsat et al., 2013). Machine learning experiments were conducted that utilized term frequency-inverse document frequency (tf-idf) features (Manning et al., 2008). Popular machine learning approaches such as NegEx (Chapman et al., 2001), Naïve Bayes and Support Vector Machines with linear kernel functions (SVM; Vapnik, 1995) were trained to recognize documents that were most likely to contain seizure terms. The Naïve Bayes and Support Vector Machines algorithms used tf-idf features while the NegEx algorithm used raw features (e.g., words) for classification of reports as ictal or non-ictal. These algorithms were seeded from 197 reports describing the occurrence of a seizure and 2,471 reports describing non-occurrence of a seizure. All three algorithms were tested on a small data set consisting of 100 reports (50 ictal and 50 non-ictal), with NegEx performing slightly better than the Naïve Bayes and SVM classifiers.

The classification of reports using NegEx was performed using a regular expression rule-based approach. The regular expression labels were selected based on negation (NEG), context (CNTX), and affirmation (AFFR). The negation labels were selected based on three different types of negations: pre-negation (PREN; i.e., did not experience), post-negation (POST; i.e., infiltrates were not shown) and pseudo-negation (PSEU). NegEx correctly classified 99% of the reports used in our pilot study of 100 reports. When applied to 18,000 sessions in TUH EEG, 844 sessions were identified as likely to have a seizure. Of these 844 sessions, manual annotation determined that 174 sessions had actual seizures.

The second method used to triage the data was to process the data through a state of the art commercial software tool, P13 rev. B from Persyst Development Corporation. (2017). We determined that 1,388 files out of 34,698 files contained seizure events. Our third method used an experimental seizure detection system known as AutoEEG (Golmohammadi et al., 2018). This system detected seizures with high confidence in 1,466 files out of 31,645 files. Files for which both systems agreed on a seizure were given the highest priority for annotation. These automated tools agreed on 146 files, or 0.42%, of the corpus. The total number of sessions that were identified as having at least one seizure by either tool was 28.

Using these three approaches, we identified 872 sessions containing 2,582 files from the original 16,168 sessions as high-yield data, meaning they were likely to contain seizures. Our annotation team then manually annotated all the data in these sessions and found that 280 of these sessions contained actual seizure events. It is interesting to note that of the three approaches for identifying high yield data, keyword search proved to be most effective. Automated seizure detection algorithms still suffer from poor performance, especially on short duration seizure events.

## Results

The most recent release of TUSZ is v1.2.0, which was released in December 2017. It contains 315 subjects with a total of 822 sessions, of which 280 sessions contain seizures. The transcriptions are provided in two file formats: (1) LBL and (2) TSE. These files can be found along with their corresponding EDF file and a de-identified report in each session. The LBL file format is transcribed on a channel basis whereas the TSE files are transcribed on a term basis. A channel-based annotation refers to labeling of the start and end time of an event on a specific channel. A term-based annotation refers to a summarization of the channel-based annotations—all channels share the same annotation, which is an aggregation of the per-channel annotations. The annotation files with “bi” extension indicate that they contain binary classes (i.e., seizure or background).

Based on the neurologist's report and careful examination of the signal, our annotation team was able to identify the type of seizures (e.g., absence, tonic-clonic). A list of these labels is shown below:

**Table:** 

SEIZ:	Seizure		
GNSZ:	Generalized Non-Specific Seizure	TNSZ:	Tonic Seizure
FNSZ:	Focal Non-Specific Seizure	CNSZ:	Clonic Seizure
SPSZ:	Simple Partial Seizure	TCSZ:	Tonic Clonic Seizure
CPSZ:	Complex Partial Seizure	ATSZ:	Atonic Seizure
ABSZ:	Absence Seizure	MYSZ:	Myoclonic Seizure

**Figure 1:**
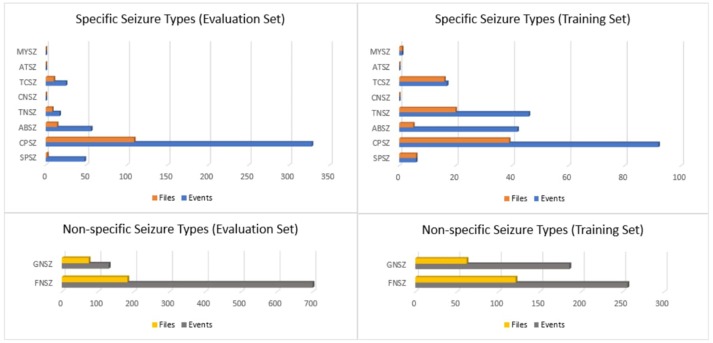
Histograms of seizure types in the TUH EEG Seizure Corpus for the evaluation and training sets.

If there was insufficient evidence to classify the type of seizure, then an event was defined as either “generalized non-specific” or “focal non-specific” depending on the focality. Histograms of the frequency of occurrence for these seizure types are shown in Figure 1.

We then segmented the data into a training and evaluation set to support technology development. The evaluation set was designed to provide a representative sampling of all conditions found in the training set under the constraint that it included 50 patients. Approximately 34% of the evaluation dataset files contain seizures, which is much higher than typical clinical EEG data. The evaluation set was designed to be compact and yet provide representative results so that it would support rapid turnaround of experiments using a moderate amount of computational resources.

**Figure 2:**
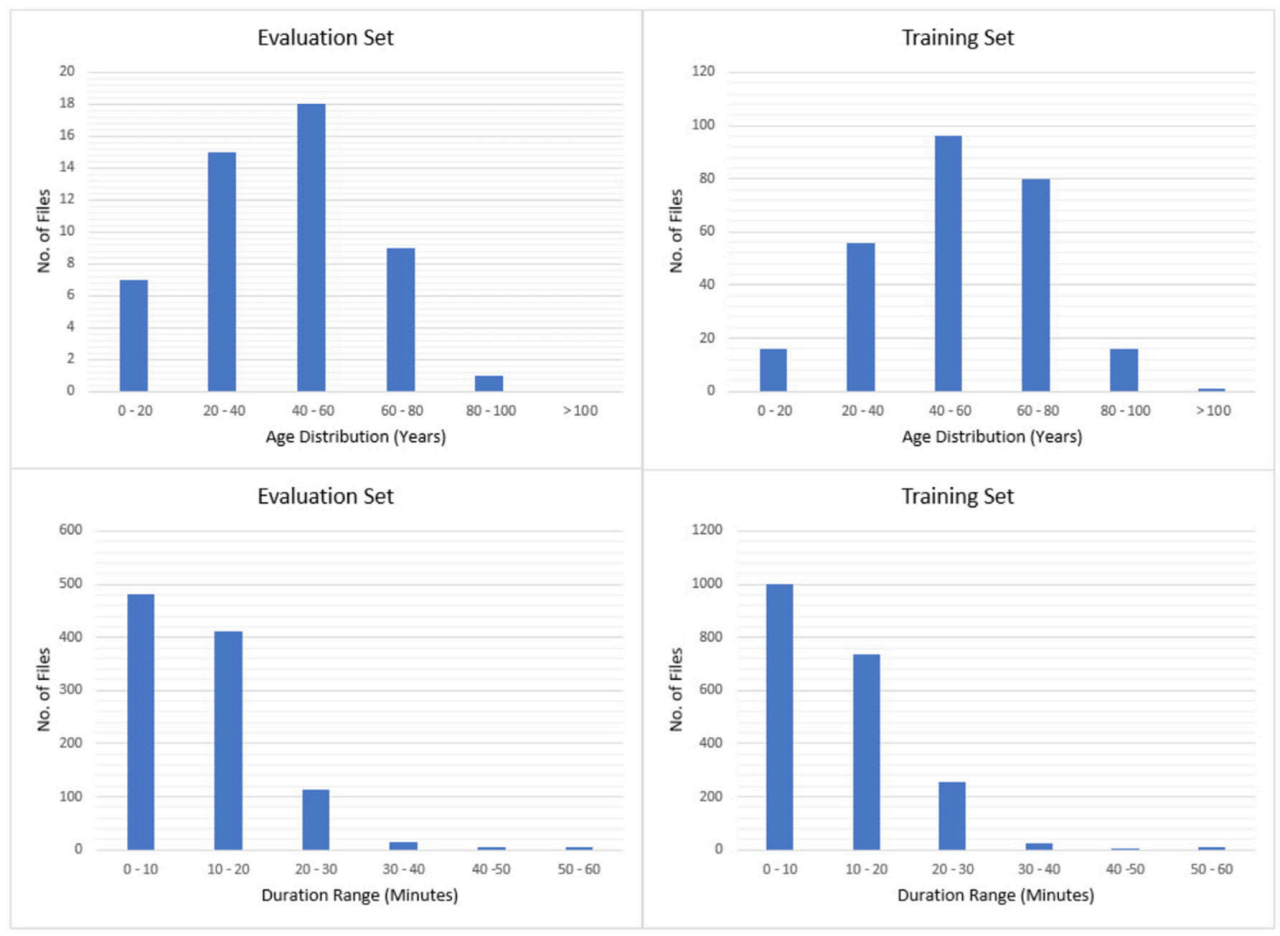
Histograms of age and duration.

The entire seizure database has been divided into training and evaluation sets to support machine learning research. All files in this corpus are pruned versions of the original EEG recordings. The duration of a single pruned file is no more than 1 h. The training and evaluation sets contain 265 and 50 subjects, respectively. The patients in the evaluation set were selected based on gender (56% of the patients in the evaluation set are female; 50.5% female in the training set) and selected to maximize a number of demographic features, as shown in Figure 2.

In addition to providing the raw signal data and annotations of seizure events, TUSZ contains metadata such as patient demographics, seizure type, and the type of EEG study. The EDF files contain the following metadata:

patient id (anonymized)
gender (male or female)
age (measured in years due to privacy issues)
recording data (DD-MMM-YYYY)
per-channel information:
  labels, sample frequency, channel physical dimension, channel physical min, channel physical max. channel digital min, channel physical max, channel prefiltering conditions

We also have released a spreadsheet with the data that describes each patient and session in terms of the following fields:

patient id (anonymized)
session id
EEG type/subtype:
  EMU/EMU (Epilepsy Monitoring Unit)
  ICU (Intensive Care Unit) /
    BURN (Burn Unit)
    CICU (Cardiac Intensive Care)
    ICU (Intensive Care Unit)
    NICU (Neuro-ICU Facility_
    NSICU (Neural Surgical ICU)
    PICU (Pediatric Intensive Care Unit)
    RICU (Respiratory Intensive Care Unit)
    SICU (Surgical Intensive Care Unit)
  Inpatient/
    ER (Emergency Room)
    OR (Operating Room)
    General
  Outpatient/Outpatient
  Unknown/Unknown (location cannot be determined)
LTM or Routine
Normal or Abnormal
Number of Seizures per Session and File
Start Time, Stop Time
Seizure Type

The EEG Type and EEG Subtype fields are used to identify the general location of the EEG session with the hospital. A qualitative assessment of the duration of the recording is indicated a field that indicated whether the EEG was a routine recording (typically an outpatient session lasting 30 min) or an extended long-term monitoring (LTM). The normal/abnormal classification follows the clinical criteria described by Lopez (2017).

While most researchers can work with the information about seizure events provided in the above spreadsheet, we also provide a series of label files that allow display of seizure labels in a time-aligned manner using an open source visualization and annotation tool (Capp et al., 2017).

## Discussion

For deep learning technology to address problems such as seizure detection, large amounts of annotated data are needed. TUSZ is the world's largest publicly available corpus of annotated data for seizure detection that is unencumbered. No data sharing or IRB agreements are needed to access the data. The entire database consists of over 504 h of data. Seizure events comprise about 36 h or about 7% of the data that has been annotated. Version 1.0.0 of the TUH EEG Corpus contains about 16,000 h of data. We have not completed processing all of that data for seizure events, but our estimate is that the overall yield for seizure data using the process described in this paper is 0.2%. Since we are accessing pruned EEGs, the overall yield from continuous data is even smaller. This is a quite sobering statistic since it reveals the challenges in building the big data resources necessary to fuel deep learning research. Accurate triaging of the data is critical to building these resources in a cost-effective manner.

TUSZ contains a rich variety of seizure morphologies. Variation in onset and termination, frequency and amplitude, and locality and focality protect the evaluation and training sets against bias toward one type of seizure morphology. Models trained using this database will be strengthened by the mix of obvious and subtle seizure morphologies and will have the potential to be better prepared for applications handling real world data. Although, part of the sampling process of the seizures could be somewhat biased by our seizure detection models, our results seem to be consistent across a wide variety of statistical models that we have run internally in our research.

Seizures are a biological process that build gradually, often lacking discrete start and stop times. Event-based and term-based annotations are therefore included in our corpus in an effort to represent two different views of seizure evolution and duration. Event-based annotations are per-channel annotations and give users a very detailed account of where in the brain the seizure originates, how it spreads, and how it terminates while term-based annotations are the same on every channel and simply include the earliest seizure start time and the latest seizure end time. Both multi-class and bi-class annotations are useful for machine learning research. Multi-class annotations provide users more specific data on the type of seizure that is occurring, while bi-class annotations simply answer the question: is there a seizure occurring or not?

We are working continuously to improve expert knowledge of seizures that can be directly channeled into improving and expanding TUSZ. Development of annotation skills and increased use of automation will allow us to continue to improve the corpus. We have developed methods to automatically annotate other events, such as eye movements, generalized periodic discharges (GPD), periodic lateralized discharges (PLD), spikes, and sharp wave (Harati et al., 2015). We have also developed methods for cohort retrieval (Obeid et al., 2016, 2017) and parsing of EEG reports (Harabagiu and Goodwin, 2016). Though our focus is currently on seizure annotation, we will soon release more metadata related to TUSZ that will enable basic neuroscience research with the data.

TUSZ has been in beta release since late 2016 and can be downloaded from https://www.isip.piconepress.com/projects/tuh_eeg/downloads/. Users must register and provide a valid email address so that we can track usage. Users can also acquire the data by sending us a disk drive. Our rapidly growing userbase currently includes over 1,300 registered users.

## Author contributions

VS designed the database, supervised training of the annotation team, monitored inter-rater agreement, and wrote the first draft of the paper. EvW our data system coordinator who supervised the development of the annotation team and execution of the project; responsible for conducting the inter-rater agreement studies and overall quality control of the transcriptions. SL developed automated tools for identification of high-yield data using natural language processing. JM responsible for the development and release of the version of TUH EEG that was used in this study (v0.6.0). LV responsible for data collection at Temple Hospital for TUH EEG (v1.0.0); paired reports with EEG sessions and contributed to the identification of high-yield data. MG developed automated seizure detection technology that was used to analyze EEG data for high-yield data; conducted machine learning experiments on the data to ensure that the final corpus was relevant; contributed to quality control of the final data. IO faculty advisor and senior co-PI on the project; responsible for funding of the project and supervised the development of the manuscript. JP faculty advisor and senior PI on the project; supervised all aspects of the project and co-wrote the manuscript.

### Conflict of interest statement

SL was employed by company Blackfynn, Inc. and MG was employed by company BioSignal Analytics. The remaining authors declare that the research was conducted in the absence of any commercial or financial relationships that could be construed as a potential conflict of interest.
